# Editorial: Advances in diagnosis and surgical treatment of benign diseases of the colon

**DOI:** 10.3389/fsurg.2026.1925043

**Published:** 2026-08-03

**Authors:** Sophia Tsokkou, Arcot Venkatasubramaniam, Vasileios Papadopoulos, Alexandros Giakoustidis

**Affiliations:** 1 First Department of Surgery, General Hospital Papageorgiou, Aristotle University of Thessaloniki, Thessaloniki, Greece; 2 Rectal Cancer Unit, Colorectal Surgery, Basingstoke and North Hampshire Hospital, Basingstoke, United Kingdom

**Keywords:** benign diseases, colon, diagnosis, surgical advances, surgical treatment

Although colorectal malignancy dominates scientific discourse and continues to attract the majority of clinical attention, the reality is that benign diseases of the colon form an equally consequential, yet often overlooked spectrum of pathology. In other words it can be refered as the '*silent killer'* of the quality of life in affected individuals. The spectrum is broad, heterogeneous, and functionally disruptive, encompassing entities that range from chronic constipation and inflammatory conditions to structural abnormalities, mucosal disorders, and rare mesothelial or vascular lesions. In many ways, benign colorectal disease functions as an *umbrella category*, sheltering a multitude of subtypes that interfere with patient's everyday lives through pain, obstruction, bleeding, altered bowel habits, nutritional compromise, and profound reductions in quality of life. Unlike cancer, where diagnostic pathways and therapeutic algorithms are well-established, benign conditions often present with diagnostic ambiguity, unpredictable trajectories, and management strategies that vary widely across institutions. As a result, the true, clinical, psychological, and socioeconomic burden remains underestimated and underevaluated, despite their daily encounterment in surgical practice.

Notably, this imbalance is compounded by a striking funding and research disparity: while colorectal cancer attracts substantial research funding and academic attention, benign colorectal pathologies receive comparatively little support, despite their considerable impact on healthcare systems and patient quality of life.

This special issue, “Advances in Diagnosis and Surgical Treatment of Benign Diseases of the Colon,*”* brings together contemporary research highlighting the critical strides made in this evolving field.

## Chronic constipation as a driver of pathology

A unifying theme threading through this issue is chronic constipation; not merely a symptom but a pivotal driver of pathology. Bai et al. present a striking case of an 85-year-old patient in whom long-standing constipation precipitated a cascade culminating in melanosis coli complicated by pneumatosis intestinalis-induced sigmoid volvulus. The meticulous clinicopathological analysis performed illustrates how chronic constipation, anthraquinone-based laxatives, and α-glucosidase inhibitors can converge to weaken the intestinal wall, promote intramural gas formation, and ultimately precipitate volvulus. It should be noted, however, that the direction of causation between pneumatosis intestinalis (PI) and volvulus warrants careful consideration. As PI most often develops secondary to raised intraluminal pressure and mucosal disruption arising from bowel obstruction and ischaemia, and given that this patient's chronic constipation and advanced age are themselves well-established risk factors for sigmoid volvulus, it is plausible that the volvulus contributed to the development of PI rather than the reverse. The fatal outcome, preceded by a three-day delay in surgical consent, highlights the urgency of clear, structured communication during informed consent in elderly patients with multiple comorbidities, and their call for enhanced surveillance in high-risk populations deserves wide clinical attention. Beyond its rarity, this case highlights a broader message: “benign mucosal changes can coexist with, and even predispose to, life-threatening complication”, challenging the assumption that “benign” equates to “low-risk.” The author's emphasis on early surgical intervention in the presence of pneumatosis and obstruction is a timely reminder of the importance of decisive operative management in frail, multimorbid patients.

Chronic constipation remains one of the most common benign colorectal complaints, yet its underlying mechanisms are often underappreciated. Shu et al. contribute an important epidemiological analysis demonstrating a positive association between the neutrophil percentage-to-albumin ratio (NPAR) and chronic constipation using NHANES data. The results add to a growing body of evidence that constipation is not purely a motility problem but may also be driven, in part, by systemic inflammation. Finding that NPAR could serve as a biomarker, especially in patients with diabetes, smokers, and those with cardiovascular disease, points to new directions for risk stratification and for understanding the mechanisms at play.

## The ileostoma as a model of intestinal biology

A comprehensive review, by Li et al. reframe the ileostomy not merely as a protective diversion but as a unique experimental and clinical window into intestinal biology. The synthesis of mucosal atrophy, neuromuscular remodeling, microbiome shifts, and endocrine alterations in the defunctioned ileum provides a compelling argument for considering the ileostoma as a model system. Particularly noteworthy is discussion of the author's of how ileostomy-induced microbial and metabolic changes may influence the efficacy of immune checkpoint inhibitors, an emerging intersection between colorectal surgery and immunotherapy. The author's exploration of interventions such as short-chain fatty acids, autologous chyme reinfusion, and early closure offers practical avenues for improving postoperative outcomes.

## Diagnostic complexity and rare presentations

Additionally, the case report by Liu et al. highlights the diagnostic complexity of *adult intussusception*, particularly in a patient with a history of rectal cancer. The case report further strenghthens a key surgical principle; unlike pediatric intussusception, adult presentations almost always require operative management due to the high likelihood of an underlying pathological lead point. The identification of a second primary ileocecal adenocarcinoma rather than recurrence, illustrates the importance of maintaining diagnostic breadth in cancer survivors. The decision to perform a right hemicolectomy with preventive fixation of the descending colon shows operative planning adapted to the patient's anatomy and recurrence risk.

The case reported by Wang et al. focuses on multiple extragenital adenomatoid tumors involving the rectum and small intestinal mesentery, which is an exceptionally rare presentation. The author's detailed radiologic and histopathologic characterization enriches the limited literature available on mesothelial-derived tumors outside the reproductive tract. The author's highlight imaging features such as cystic-solid mass morphology, peripheral enhancement, and calcifications that may help distinguish these benign lesions from malignant mesothelioma, lymphangioma, or metastatic disease. This is a reminder to clinicians that benign tumors can mimic aggressive pathology and that accurate diagnosis prevents unnecessary radical treatment.

## Research trends in inflammatory bowel disease surgery

Finally, Xue et al. provides a bibliometric analysis to chart the main research trends in inflammatory bowel disease surgery across the global literature. The VOSviewer analysis points to the United States, United Kingdom, and Italy as the top contributors, with the University of Toronto emerging as a leading institution. The identification of five major thematic clusters, including robotic surgery, postoperative recurrence, biologic therapy, and quality-of-life outcomes, highlights the breadth of contemporary IBD surgical research. This work stresses the need for sustained international collaboration and the importance of integrating surgical innovation with evolving medical therapies.

## Conclusions

The contributions in this Special Issue emphasize a fundamental principle: the care of benign colonic illnesses requires a tailored, multidisciplinary, and profoundly patient-centered approach. As surgical procedures become increasingly advanced and diagnostic tools more accurate, our obligations extend well beyond the operating theatre. The true measure of advancement resides in the continuous provision of care, from prompt and accurate diagnosis, to enhanced preoperative planning, to meticulous surgical execution, and ultimately to ongoing, considerate postoperative follow-up that reinstates function and quality of life.

